# Perspectives on the Market Globalization of Korean Herbal Manufacturers: A Company-Based Survey

**DOI:** 10.1155/2015/515328

**Published:** 2015-06-14

**Authors:** Dongsu Kim, Miyoung Ahn, Jeeyoun Jung, Soohyun Kwon, Eun-Ji Park, Ki Hoon Koo, Jong-Min Woo

**Affiliations:** ^1^Policy Division, Korea Institute of Oriental Medicine, 1672 Yuseongdae-ro, Yuseong-gu, Daejeon 305-811, Republic of Korea; ^2^School of Korea Medicine, Pusan National University, Yangsan 626-870, Republic of Korea; ^3^Clinical Research Division, Korea Institute of Oriental Medicine, 1672 Yuseongdae-ro, Yuseong-gu, Daejeon 305-811, Republic of Korea; ^4^Department of Convergence Technology Evaluation Researcher, Korea Technology & Information Promotion Agency for SMEs, 593 Daedeok-daero, Yuseong-gu, Daejeon, 305-340, Republic of Korea

## Abstract

The growth of herbal markets has increased substantially in South Korea, but the worldwide market share remains small despite significant governmental efforts. This study aimed to characterize manufacturing employment and identify employees' general perceptions of market expansion. A survey study covering 567 companies was conducted using face-to-face interviews in 2012. Data were analyzed using comparisons among three manufacturing groups (i.e., the herbal dietary supplement manufacturing group, the herbal medicine manufacturing group, and the personal care product manufacturing group) or among the manufacturers themselves. We found that the majority of the manufacturing employee respondents were regular permanent and production workers. The domestic distributors were primarily chain stores/direct outlets or retailers/wholesalers, and the dominant product was red ginseng (*hongsam*). Although the responding companies exhibited a variety of perspectives, “advertisement/public relations” was cited as the most important factor in the development of the herbal industry. In contrast, “low manpower/seeking business partners” were the most crucial limiting and challenging factors for market globalization. Our results can be used to design a proper national plan by reducing the gaps in perspective between herbal product producers and policy makers.

## 1. Introduction

Nature has been a great source of medicinal substances, and in most countries, individuals have used herbal products for thousands of years [1]. Despite an exponential increase in the use of herbal products, scientists have investigated only 5% to 15% of the approximately 250,000 species of higher plants [2, 3]. During the past decade, there has been increasing public interest and acceptance of herbal products in both developing and developed countries. This acceptance has been attributed to an increasing recognition that these products are generally nontoxic, have few side effects on average, are compatible with physiological flora, and are available at affordable prices [3, 4].

The consumption of herbal medicines has increased markedly worldwide. In China, the herbal industry has experienced a surge in recent decades because of concerted efforts to globalize the country's botanical medicines. Chinese herbal medicines such as* mahuang* and* ginko* have successfully entered the international market [5], and their use is increasing worldwide. In the US, herbal and dietary supplements (HDS) represent a $180 billion market, and in 2004, 18.9% of individuals reported using them [6]. American consumers spend approximately $6 billion on HDS annually [7]. The world market for herbal medicine, including herbal products and raw materials, has been estimated to have an annual growth rate between 5% and 15%, and approximately 30% of international drug sales are derived from botanical products [8–10]. It is forecast that, by the year 2050, the total global herbal drug market will increase to US $5 trillion [11].

In January 2000, Korea's Congress enacted* The Promotion of the Research and Development of Wonder Drugs Using Natural Substances Act*. This act facilitates scientific investigations using natural substances.* The Korean Medicine and Pharmaceutics Promotion Act* was enacted in August 2004 and promotes research and development (R&D) and industry in the field of traditional Korean medicine (TKM). As a result, the growth of the herbal industry has dramatically accelerated in terms of both herbal technologies and domestic market size. However, the industry's global share remains small. Thus, more comprehensive approaches are required to establish an efficient company-based strategy for integration into the global market.

The primary purpose of this study was to characterize the workforce in the herbal manufacturing industry and investigate manufacturers' perceptions of the limiting/challenging factors for market globalization. We found that “advertisement/public relations” was regarded as the most important factor for the development of the herbal industry. To eliminate strong dependence on the domestic market, finding business partners was also necessary. It is worthwhile to establish an effective national plan for herbal marketing based on the demands, perceptions, and perspectives of the industry's workforce.

## 2. Methods

### 2.1. Questionnaire

The survey instrument included two major sections. One section consisted of questions regarding workforce characteristics, such as manufacturing product type, employee occupational status, product sources, and domestic vendors. The other section consisted of questions about perceptions of market expansion, such as important or limiting factors with respect to the development of the herbal market and strategies for market globalization. To ensure the accuracy of the data that were collected, certain questions were designed to allow multiple answers. To establish the content validity of our survey, we consulted an expert panel. A draft of the questionnaire was pilot-tested with relevant stakeholders until each domain was completely captured via a repetitive adjustment based on our panel's reviews. Prior to conducting the survey, we retested whether the questions were apparent, understandable, and presented in a logical order (Additional File 1 in Supplementary Material available online at http://dx.doi.org/10.1155/2015/515328). The survey and the study method were approved by the ethical review committee of the Korea Institute of Oriental Medicine (KIOM), located in South Korea.

### 2.2. Participants

Five hundred sixty-seven herbal manufacturing firms were randomly selected based on Neyman's optimum allocation method. The participants were recommended by their companies as well-qualified employees, that is, employees with professional oversight who had been working for several years and had knowledgeable insight into the company's business structures. The responding manufacturers were categorized into three groups according to their product specificities: herbal dietary supplements (i.e., ginseng products, herbal drinks, and herb extracts), herbal medicine (traditional herbal medicine and conventional herbal medicines), and personal care products (herbal cosmetics and herbal sanitizers). We conducted face-to-face interviews with an appointed employee from each company between January 12 and August 24, 2012, in South Korea. As previously mentioned, one designated person per company was interviewed with our instrument at the workplace. Participants who were willing to participate in the survey provided informed consent. Data were collected from the interviewed individuals by a professional survey company.

### 2.3. Definitions

In this paper, the herbal companies were defined and classified according to the ninth Korean Standard Industrial Classification (KSIC-9) with several modifications [12]. In brief, ginseng product manufacturers (KSIC-9 code: 10795) were defined as companies that produce ginseng goods using raw or processed ginseng materials. Herbal drink manufacturers (KSIC-9 code: 11119, 11129, 10709, and 11209) are companies that produce tea or alcoholic or nonalcoholic beverages using herb materials. Herb extract manufacturers (KSIC-9 code: 10797 and 10749) are companies that produce crude additives, liquefied additives, or health-functional foods by processing natural herbs. Traditional herbal medicine manufacturers (KSIC-9 code: 21220) produce standardized herbal medicines or herbal remedies based on traditional Korean knowledge and literature. Conventional herbal medicine manufacturers (KSIC-9 code: 21210) produce labeled herbal medicines by processing a raw herb or its dried extracts into different forms, including tablets, capsules, syrups, essential oils, and ointments. Herbal cosmetics manufacturers (KSIC-9 code: 20433) produce beauty goods, such as perfumes, beauty washes, cosmetics, or related products. Herbal sanitizer manufacturers (KSIC-9 code: 20432) produce a variety of sanitary items, including detergents, soaps, toothpastes, or cleansers, by adding herbal ingredients.

### 2.4. Statistical Analysis

All of the data collected by the survey were analyzed using the Statistical Package for Social Sciences (SPSS Inc., Chicago, IL) v.18.0. Data were assessed using a Pearson's chi-square test to compare categorical variables (*P* = 0.05).

## 3. Results

### 3.1. Characteristics of the Manufacturers' Workers

Table 1 shows the characteristics of the workforce of the responding manufacturers. The gender composition of each manufacturer was largely equal, with the exception of the conventional herbal medicine manufacturers. The workers' occupational types varied, but the vast majority were “production workers.” Conventional herbal medicine manufacturers exhibited a relatively high rate of “researchers” (19.9%) compared to other manufacturers. The workforce of ginseng product manufacturers consisted of only 4.1% researchers, which was the lowest rate among all of the respondents. Many of the workers were regular, permanent employees. This status was particularly noticeable in the herbal medicine-manufacturing group (87.9% in traditional herbal medicine manufacturers; 98.8% in conventional herbal medicine manufacturers). In contrast, the proportion of workers who were permanent employees was lower in the dietary supplement-manufacturing group (65.4% in ginseng product manufacturers; 72.0% in herbal drink manufacturers; 76.9% in herb extract manufacturers). Regarding occupational status, most conventional herbal medicine manufacturers appeared to offer steady employment and were operated based on worker manpower. In comparison, a large number of ginseng product manufacturers operated private businesses and did not frequently hire outside employees. Such manufacturers were occasionally aided by their families.

### 3.2. Domestic Markets for Herbal Manufacturers

**Figure 1:**
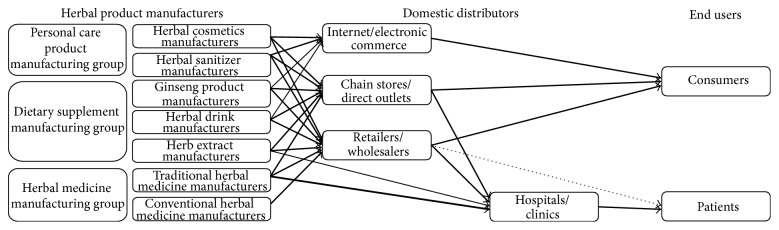
Domestic supply chain of herbal products in South Korea. The arrow depth represents the proportion of product supply for each distributor. The dotted arrow indicates over-the-counter (OTC) drug supply.

The survey asked about the domestic distributors for each manufacturer's products.  Table 2 shows that the primary domestic markets of the respondents were chain stores/direct outlets and retailers/wholesalers. The percentages of chain stores/direct outlets were highest among ginseng product manufacturers (75.1%), and the percentages of retailers/wholesalers were highest among conventional herbal medicine manufacturers (88.9%). The percentages of hospitals/clinics were highest among traditional herbal medicine manufacturers (33.6%). The ratio of the internet/electronic commerce market was lowest in the herbal medicine-manufacturing group (traditional herbal medicine manufacturers: 2.2%, conventional herbal medicine manufacturers: 0%). As a result of the survey, a domestic supply chain for herbal products could be depicted (Figure 1). Regardless of the herbal manufacturing group, retailers/wholesalers and chain stores/direct outlets were the dominant markets in South Korea.

The responding companies were also asked which herbal species were commonly used as product sources. Red ginseng (*hongsam*), which is produced by steaming fresh ginseng, was clearly predominant among ginseng product manufacturers, herbal drink manufacturers, and herb extract manufacturers. It was also overwhelmingly predominant among ginseng product manufacturers (90.0%). Among herbal drink manufacturers,* bokbunja* was the second-largest herbal source (16.9%), followed by* omija* (10.4%),* sansuyu* (7.8%), and* ogapi* (6.5%).* Rehmanniae radix* (*jiwhang*) was a top-ranked common source for traditional medicine manufacturers (12.8%). However, its prominence was not substantially greater than those of other sources:* hongsam* (10.6%),* gamcho* (10.6%), and* dangwi* (10.6%) (Additional File 2). 

### 3.3. Elements for the Development of Herbal Manufacturing Industries

To assess each respondent's perception of the increase in market share, we asked which factors were most important. Overall, “advertisement/public relations” was chosen as the most important factor for herbal industry development. Notably, this response was most common in the dietary supplement manufacturing group. A total of 53.4% of ginseng product manufacturers, 46.4% of herbal drink manufacturers, and 42.0% of herbal extract manufacturers deemed it most important. Among conventional herbal medicine manufacturers, the greatest proportion of respondents rated “R&D investment (45.5%)” and “legislative support (45.5%)” as the most important factors for development, whereas the proportion that chose “public relations (9.1%)” was considerably lower. The proportion that chose “political support” was low for all respondent manufacturers (Table 3).

### 3.4. Limitations of Global Market Expansion

The respondents were asked whether they had experience with market globalization. Only approximately one-fourth of the respondents had attempted to promote their businesses abroad (data not shown). We asked the companies that had this experience what problems or difficulties they had faced. “Low manpower” was the difficulty most often reported by ginseng product manufacturers (67.7%), herbal extract manufacturers (48.8%), and herbal cosmetic manufacturers (36.2%). Additional responses that were frequently reported included “insufficient funds” (26.3%) by herbal drink manufacturers, “language problems” (40.3%) by traditional herbal medicine manufacturers, and “poor marketing strategies” (57.5%) by conventional herbal medicine manufacturers. It was interesting that “language problems” (35.1%) were considered a major interfering factor among traditional herbal medicine manufacturers. Conventional herbal medicine manufacturers were more likely to need to find foreign business partners and establish marketing channels (Table 4).

### 3.5. Challenging Factors for Global Marketing

All of the participants were asked what factors were necessary to integrate their products into global markets. As shown in Table 5, 59.3% of ginseng product manufacturers and 40.5% of herbal drink manufacturers cited “seeking business partners” as the highest-rated strategy. In contrast, 50.0% of herbal extract manufacturers cited “financial support” as the highest-rated strategy. Although “seeking business partners” was the most-cited strategy among traditional herbal medicine manufacturers (44.4%), the range of scores did not differ considerably from other responses.

### 3.6. Korean Herbal Manufacturers' Suggested Strategies for Achieving Market Globalization

**Figure 2:**
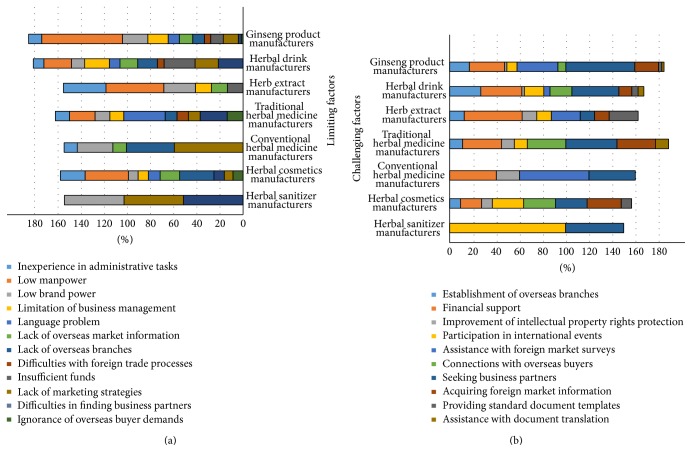
Histogram for comparison of all rated factors. On the bar, different colors represent each factor, and the length represents the response rate (%). The left side illustrates limiting factors, and the right side illustrates challenging factors.

**Figure 3:**
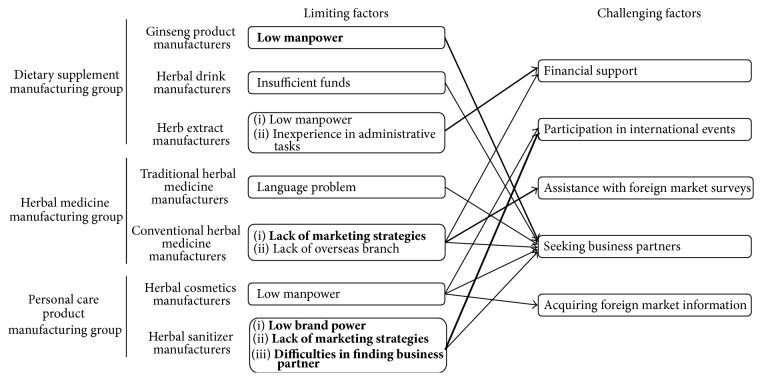
Major limiting factors and challenging factors for market globalization. Four limiting factors are in bold. The starting point of each arrow indicates the responding manufacturer. The end point of each arrow indicates the major challenging factor. The arrow depth represents the proportion of responses.

In this survey, we found outstanding demand for market globalization. With respect to limiting factors, “low manpower” was mostly cited in the dietary supplement manufacturing group and the personal care product-manufacturing group. “Lack of marketing strategies” was mostly cited in the herbal medicine-manufacturing group. With respect to challenging factors, most of the respondents cited “seeking business partners” regardless of manufacturing types (Figure 2). For our conclusion, we selected the following four major limiting factors: low manpower, lack of marketing strategies, low brand power, and lack of marketing strategies. In addition, we suggest five strategic priorities for efficient support. The most crucial factor was seeking business partners, which was followed by participation in international events, financial support, assistance with foreign market surveys, and acquiring foreign market information (Figure 3).

## 4. Discussion

Herbs and plants can be processed and administered in various ways. The forms in which herbs and plants are used include the whole herb, teas, syrups, essential oils, ointments, salves, rubs, capsules, and tablets that contain a ground or powdered form of a raw herb or its dried extracts [13]. The classification of herbal products may vary depending on the countries in which they are used. In the EU, they are classified as herbal medicines with requirements for safety and quality standards. Certain herbs may be designated as food supplements. In the US, herbal products are classified as dietary supplements or botanicals, not medicines [14]. The World Health Organization (WHO) defines “herbal medicine” as a “plant-derived material or preparation with therapeutic or other human health benefits and which contains either raw or processed ingredients from one or more plants.” Herbal medicines can be classified into three groups: raw plant material, processed plant material, and medicinal herbal products [15, 16].

With respect to domestic distributors, there was a considerable difference between conventional herbal medicinal manufacturers and other manufacturers. As shown in Figure 1, the supply chain of the conventional herbal medicine manufacturers was remarkably centralized on retailers/wholesalers (88.9%), whereas others were spread among two or more distributors. The sales channel of medicinal manufacturers was substantially influenced by national health-care policy. Until the introduction of the 2000 Korean Separation of Dispensary from Medical Practice, in which pharmacists were regulated separately from physicians, hospitals/clinics were some of the largest sales agents for conventional medicine manufacturers. Patients preferred hospital pharmacies to retail drugstores for several reasons: convenience, physician recommendation, nonstandardized prescriptions, and greater assurance of pharmaceutical quality. Manufacturer salespersons often contacted hospitals and/or doctors directly to sell their products [17]. Under the new medical system, hospitals/clinics were no longer allowed to sell drugs. Only prescribed medicines could be obtained at appointed drugstores or local pharmacies. Medicine sales via retailers/wholesalers are expected to increase more rapidly since the 2012 revised Pharmaceutical Affairs Law in South Korea authorized several over-the-counter (OCT) drugs for sale by retailers and convenience stores.

As Korea's most popular herbal dietary supplement, ginseng has been widely distributed across the country through a number of stores [18]. We found that red ginseng was the primary product in the dietary herbal manufacturing group. There is a variety of commercial ginseng products. Four representative products are red ginseng (*hongsam*), white ginseng (*baeksam*), black ginseng (*heuksam*), and wild ginseng (*sansam*). Except for wild ginseng, these products are manufactured from fresh ginseng (*susam*). Substantial differences have been reported to exist among the different types of ginseng products [19, 20]. Red ginseng is made by steaming fresh ginseng at high temperatures. It is the most popular product in the Korean ginseng markets, representing approximately 59% of the Korean market, and its consumption is steadily increasing each year in both the ginseng and health-food markets [21]. White ginseng is produced from fresh ginseng by dehydration, whereas black ginseng is produced from white ginseng by nine cycles of steaming procedures. Ginseng is manufactured for various practical purposes, such as medicine, or taken on a daily basis in the form of tea, cookies, candy, and gum. Currently, 456 companies are clustered in Geumsan, South Chungcheong Province, South Korea, accounting for 70.6% of the total number of ginseng manufacturers. Korean ginseng, usually referred to as* Goryeo Insam*, has been exported to China and Japan since approximately the 12th century and has earned fame in other countries. South Korea, China, Canada, and the US are the largest producers. Their total production of fresh ginseng is more than 99% of the total world ginseng production [21]. Although South Korea is among the world's largest producers ($38 million in 2009), the amount consumed domestically is larger than the amount exported [21].

Most synthetic chemicals can be harmful to the skin. Therefore, natural ingredients in cosmetics and sanitizers can be a useful alternative. Increasing awareness of the safety and reduced toxicity of herbal ingredients has resulted in an increase in the consumption of these products. It has been estimated that the number of US adults who use herbs to treat medical and cosmetic conditions increased from 3% in 1990 and 12% in 1997 to 21% in 2001 [22]. Korean herbal cosmetics have been developed over the last 30 to 40 years by applying ancient medical wisdom, particularly as documented in* Donguibogam*. Korean herbal cosmetics companies have released “*hanbang cosmetics*,” which are scientifically formulated with multiple herbs based on TKM. These products are best-selling items and constituted 16% of the global market share of herbal cosmetics in 2007. A large number of modern technologies are employed in making these personal-care products, and numerous multinational companies are competitively seeking proper herbal materials. In addition to the cosmetics industry, the manufacture of natural sanitizers formulated with herbal ingredients presents many opportunities for growth in parallel with increased consumer concern about the use of harsh synthetic detergents. More specifically, many individuals perceive herbal ingredients to be more environmentally favorable than synthetic ingredients. In many countries, investments are being made in roots, leaves, seeds, flowers, and stems that are believed to possess useful properties. The European personal-care market is the world's largest personal-care market. Nearly every month, new items are introduced to capture the attention of consumers who are looking for simple, natural, and environmentally friendly products. Most Korean herbal sanitizer companies are relatively small. However, their products are of high quality and competitive. Even in a saturated market, it remains possible to find a niche.

“Advertisement/public relations” was selected as the most important factor for the development of the herbal industry by the dietary supplement-manufacturing group. This view was reasonable because, given the nature of health-functional foods, market development was considered directly related to both the degree of familiarity and consumer awareness. In connection with the same question, it was interesting that the response of traditional herbal medicine manufacturers contrasted with that of conventional herbal medicine manufacturers. The former responded “advertisement/public relations,” whereas the latter responded “R&D investment” and “legislative support.” There was a significant difference between the two manufacturers. Traditional herbal medicine manufacturers were established to produce modernized herbal medicines with well-known materials by mixing or boiling according to the traditional manner. These companies made significant efforts to make their products attractive enough to be voluntarily purchased instead of competing medicines. In this respect, sales volume was strongly influenced by brand familiarity. In contrast, conventional herbal medicine manufacturing was established to produce novel drugs by isolating or extracting specific chemical substances from any type of herb. To complete drug development, numerous repetitive experiments were performed, from the laboratory bench to clinical bedside studies. Therefore, it was inevitable that substantial R&D funding would be required to demonstrate the efficacy and safety of the new substances. Subsequently, these companies encountered numerous complicated regulatory issues that required resolution prior to approval from the Ministry of Food and Drug Safety (MFDS), formerly known as the Korea Food and Drug Administration (KFDA). Therefore, these companies were likely to cite “R&D investment” and strong “legislative support” as important factors.

Most Korean companies in the dietary supplement group were small, had been established on a small scale with limited capital, and featured a limited number of products compared with companies in the other groups. Their sales relied on domestic consumption. Even among their ginseng products, more than 80% of total production was sold in domestic markets. The primary reasons cited by these small companies for not expanding their markets were insufficient manpower, a lack of money, and limitations on business management. Therefore, these companies had a strong tendency to claim a need for financial support. In contrast, conventional herbal medicine manufacturers were generally established with sufficient capital and an adequate number of items. These companies pursued overseas ventures and hoped to establish a global reputation using an advanced strategy. In comparison, traditional herbal medicine manufacturers adopted a unique perspective. They most frequently reported “language problems” as a critical difficulty in global marketing. As previously mentioned, we presumed that these problems may have resulted from the difficulty of translating Chinese characters, which were originally used to name and describe the herbs, into English, an international language. Nearly all Korean traditional literature is written in Chinese characters, and therefore the vast majority of traditional herbal medicines are referred to using Chinese words; this means that traditional herbal medicine manufacturers need to acquire translation skills prior to entering the international market. However, it was notable and unexpected that they cited “seeking business partners” (44.4%), “connection with overseas buyers” (33.3%), and “financial support” (33.3%) as strategic points with respect to global marketing. This disparity may result from different viewpoints. These manufacturers may consider poor language skills to be a problem that should be solved not systemically but personally. Interestingly, all of the herbal sanitizer manufacturer respondents believed that “participation in international events” was necessary, which suggests that they expect international events to provide opportunities for marketing and introducing their brands.

Traditional herbal medicines are based on classic Korean literature. Manufacturers who want MFDS approval should specify an exact reference from the cited traditional literature. Strictly speaking, these products are newly processed drugs rather than newly created medicines and involve both known botanical materials and modern technology. In 2009, these manufacturers earned US $201 million, an increase of 20.6% over earlier years. Among them,* Kwangdong Pharmaceutical Co. Ltd.*, the largest Korean traditional herbal medicine manufacturer, made approximately US $22 million in one year. However, this amount was less than approximately 1/150th of China's* Beijing Tong Ren Tang Chinese Medicine Co. Ltd*. and approximately one-tenth of Japan's* Tsumura Pharmaceuticals Co. Ltd*. Unlike traditional herbal medicines, conventional herbal medicines are fundamentally based on modern experimentation. They are new drugs that contain various phytochemical compounds derived from multiple botanicals. The world market for conventional herbal medicines was estimated at US $89 billion in 2013. Sales of* Tamiflu*, an antiviral drug derived from Chinese star anise, have ballooned since the outbreak of swine flu in 2009, bolstering the profits of the world's largest anticancer drug maker,* Roche*. Sales of* Tamiflu* accounted for 3.2 billion Swiss Francs that same year. Korean conventional herbal manufacturers earned US $182 million in 2013, similar to earlier years but representing a 15.4% increase over 2008.* Stillen*, a gastric mucosal drug derived from* Artemisia asiatica* by* Dong-A ST Co. Ltd*., is a representative South Korean herbal drug. Its sales accounted for US $82.6 million in 2009 and strongly anticipated a continuation in the upward trend in sales.

Global companies develop internationally competitive products, successfully penetrate overseas markets, and continually attempt to extend their businesses abroad. It is true that most of the Korean herbal industry has been inferior to developed countries. However, there are obvious opportunities for market expansion. First, Korea has many indigenous plant resources. The Korean Peninsula is home to approximately 4,600 plant species. Moreover, almost all of Korea's plants are preserved in well-equipped national storage locations: in 2014, 1,617 species were kept in the Rural Development Administration (RDA) and 2,432 species were kept by the Korea Forest Service (KFS). Abundant resources are a latent repository of herbal products. Second, Korea has a great deal of traditional knowledge about herbs and has developed medical practices for many years, resulting in valuable classic medical publications such as* donguibogam*,* hyangyakjipseongbang*,* euibangyoochui*, and* donguisusebowon*. Specifically,* donguibogam* was the first book on medicine to be registered in the Memory of the World Register by UNESCO in 2009. These books detail methods for using medicinal plants to create remedies and medications. With Korea's world-class heritage and great wealth of clinical experience, the potential for growth in the Korean herbal industry is enormous. Third, the Korean government has pursued a solid policy for nurturing the traditional-medicine industry, including the herbal industry. To reduce the gap between the global companies and Korean companies, the government developed the 2008–2015 National Korean Traditional Medicine Development Plan, which focuses on both R&D promotion and the flourishing of the industry. During this period, the Korean government invested US $1 billion. In addition, since 2013, the government has begun a TKM promotion policy under the slogan “Globalization of Traditional Korean Medicine,” which aims to fulfill a 5% market share in the international CAM market by 2017. To ensure TKM's public relations worldwide, a diverse array of government-driven activities have been implemented. These activities include hosting international exhibitions and conferences such as the International Congress on Complementary Medicine Research (ICCMR) 2015, supporting charitable work such as Official Development Assistance (ODA) and engaging in regulatory improvement of foreign patient care programs on TKM.

This survey has several limitations. First, our results do not represent all workers' viewpoints. Because of the survey's small sample size, particularly among conventional herbal manufacturers and herbal sanitizer manufacturers, it is difficult to clearly define these manufacturers' perceptions. Second, although the companies that participated in this study were selected randomly, the respondents we contacted were appointed by their companies. Therefore, the findings may have been affected by selection bias. Third, the answers to several questions included many options, particularly with respect to “difficulties for global marketing” (Table 4) and “suggested strategies for market globalization” (Table 5). Therefore, the answers to these questions were both varied and decentralized. Fourth, our results were slanted toward small-sized manufacturers. We did not divide the respondents into small and medium-sized enterprises (SMEs) and large enterprises. Because the ratio of SME manufacturers included 97% of the respondents, a minority of large manufacturers' perceptions were not apparent. After completing our analyses, we asked for and analyzed minority opinions again with some representative Korean herbal manufacturers (*N* = 15) that employed more than 50 workers. Forty percent of respondents in the large dietary supplementary manufacturer group cited “inexperience in administrative tasks” as the highest-rated limitation for market globalization. The ratio for citing “seeking business partners” as the challenging factor was either similar or a bit higher (Additional File 3). As a result, future surveys that include more workers from diverse manufacturers are required.

## 5. Conclusion

Based on a survey of 567 manufacturers in Korea, we discovered that Korean herbal manufacturers have varying perspectives on market expansion. Nevertheless, we were able to identify the crucial demands and major perceptions of market globalization. This study represents the first attempt to analyze these problems based on the views of individuals who work in the field. This investigation is meaningful because herbal product manufacturing is a promising business, and market globalization is in the national interest. Our results can be used to design a suitable national plan by diminishing the gaps in perspectives between herbal product makers and policy makers. For continued growth and development in the herbal industry, it is important for Korean brands to be recognized by the market and consumers alike.

## Supplementary Material

Additional file 1: Questionnaire on the perceptions of market expansion and globalization. The survey instrument was designed to include two major sections. One section consisted of the questions regarding the characteristics of workforce. The other section consisted of the questions regarding the perception on the market expansion and strategies for market globalization.
Additional file 2: Herbal items produced by respondent manufacturers. The respondents were asked what herbal species were commonly used as product sources. Red ginseng was predominant in ginseng product manufacturers, herbal drink manufacturers, and herb extract manufacturers.
Additional file 3: Fifteen representative Korean herbal manufacturers' perceptions of market globalization. Forty percent of respondents in the large dietary supplementary manufacturer group cited “inexperience in administrative tasks” as the highest-rated limitation for market globalization. Meanwhile, the ratio for citing “Seeking business partners” as the challenging factor was either similar or a bit higher.

## Figures and Tables

**Table 1:** Manufacturing workers' occupational characteristics.

Manufacturers	Company no.	Gender (%)		Occupational type (%)			Occupational status (%)					
		Male	Female	Researcher	Production worker	Office worker and others	Regular permanent worker	Temporary daily worker	Self-employed worker	Unpaid family worker	Fixed-term worker	Others
*Dietary supplement manufacturing group *												
Ginseng product manufacturers	186	46.3	53.7	4.1	62.8	33.1	65.4	9.3	15.2	8.2	1.9	0.0
Herbal drink manufacturers	108	53.8	46.2	5.3	71.2	23.5	72.0	25.2	1.1	1.6	0.0	0.1
Herb extract manufacturers	64	54.2	45.8	12.0	69.3	18.7	76.9	19.7	1.6	1.7	0.0	0.0
*Herbal medicine manufacturing group *												
Traditional herbal medicine manufacturers	111	47.2	52.8	8.5	60.8	30.7	87.9	4.1	4.9	3.0	0.0	0.1
Conventional herbal medicine manufacturers	12	76.8	23.2	19.9	46.4	33.8	98.8	1.2	0.0	0.0	0.0	0.0
*Personal care product manufacturing group *												
Herbal cosmetics manufacturers	65	46.3	53.7	15.8	54.9	29.3	91.2	7.1	0.2	0.5	1.0	0.0
Herbal sanitizer manufacturers	21	51.2	48.8	7.9	58.8	33.3	85.5	0.0	7.9	6.6	0.0	0.0

*Note*. The values are the percentages of participants.

**Table 2:** Domestic distributors for herbal manufacturers.

Manufacturers	Company no.	Hospitals/clinics	Retailers/wholesaler	Chain stores/direct outlets	Internet/electronic commerce	Others
*Dietary supplement manufacturing group *						
Ginseng product manufacturers	186	2 (1.1)	101 (54.5)	140 (75.1)	19 (10.1)	4 (2.1)
Herbal drink manufacturers	108	2 (1.8)	72 (66.4)	52 (48.2)	18 (16.4)	13 (11.8)
Herb extract manufacturers	64	8 (12.8)	34 (53.2)	31 (48.9)	5 (8.5)	4 (6.4)
*Herbal medicine manufacturing group *						
Traditional herbal medicine manufacturers	111	370 (33.6)	33 (29.9)	56 (50.4)	2 (2.2)	4 (3.6)
Conventional herbal medicine manufacturers	12	1 (11.1)	11 (88.9)	1 (11.1)	0 (0.0)	4 (33.3)
*Personal care product manufacturing group *						
Herbal cosmetics manufacturers	65	4 (5.4)	32 (48.6)	23 (35.1)	19 (29.7)	14 (21.6)
Herbal sanitizer manufacturers	21	0 (0.0)	8 (40.0)	10 (50.0)	6 (30.0)	2 (10.0)

*Note*. The questions allowed for multiple answers. The values are the numbers (percentages) of participants.

**Table 3:** Perceived important elements for the development of Korea's herbal industries.

Manufacturers	Company no.	Trust-building	Political support	R&D investment	Advertisement/public relations	Legislative support	*P* value^*∗*^
*Dietary supplement manufacturing group *							
Ginseng product manufacturers	186	79 (42.3)	92 (49.2)	46 (24.9)	99 (53.4)	12 (6.3)	**0.0048**
Herbal drink manufacturers	108	33 (30.9)	36 (33.6)	41 (38.2)	50 (46.4)	13 (11.8)	
Herb extract manufacturers	64	17 (26.0)	18 (28.0)	23 (36.0)	27 (42.0)	0 (0.0)	
*Herbal medicine manufacturing group *							
Traditional herbal medicine manufacturers	111	30 (26.6)	35 (31.7)	28 (25.2)	48 (43.2)	16 (14.4)	**0.0395**
Conventional herbal medicine manufacturers	12	4 (36.4)	2 (18.2)	5 (45.5)	1 (9.1)	5 (45.5)	
*Personal care product manufacturing group *							
Herbal cosmetics manufacturers	65	28 (43.6)	13 (20.5)	27 (41.0)	23 (35.9)	5 (7.7)	0.4987
Herbal sanitizer manufacturers	21	4 (18.2)	2 (9.1)	10 (45.5)	8 (36.4)	2 (9.1)	

*Note*. The questions allowed for multiple answers. The values are the numbers (percentages) of participants.

^*∗*^ *P* value using chi-squared test or Fisher's exact test for categorical variables; *P* values with statistical significance are presented in bold.

**Table 4:** Respondent-cited difficulties that were experienced during preparation for global marketing.

Manufacturers	Company no.	Inexperience in administrative tasks	Low manpower	Low brand power	Limitation of business management	Language problems	Lack of overseas market information	Lack of overseas branches	Difficulties with foreign trade processes	Insufficient funds	Lack of marketing strategies	Difficulties in finding business partners	Ignorance of overseas buyer demands	Other	*P* value^*∗*^
*Dietary supplement manufacturing group *															
Ginseng product manufacturers	186	20 (11.0)	126 (67.7)	40 (21.5)	32 (17.3)	17 (9.1)	21 (11.3)	19 (10.3)	9 (5.1)	19 (10.4)	23 (12.4)	5 (2.5)	2 (1.1)	0 (0.0)	**<0.0001**
Herbal drink manufacturers	108	10 (8.9)	25 (23.2)	12 (10.9)	23 (20.9)	10 (9.0)	16 (15.0)	17 (16.2)	6 (5.4)	28 (26.3)	21 (19.4)	22 (20.6)	0 (0.0)	0 (0.0)	
Herb extract manufacturers	64	23 (35.6)	31 (48.8)	17 (26.5)	8 (13.2)	0 (0.0)	8 (13.2)	0 (0.0)	0 (0.0)	8 (13.2)	0 (0.0)	0 (0.0)	0 (0.0)	0 (0.0)	
*Herbal medicine manufacturing group *															
Traditional herbal medicine manufacturers	111	13 (11.7)	24 (21.4)	14 (12.6)	13 (11.7)	39 (35.1)	0 (0.0)	11 (9.7)	11 (9.7)	0 (0.0)	10 (9.4)	26 (23.4)	14 (12.6)	10 (9.4)	
Conventional herbal medicine manufacturers	12	1 (11.2)	0 (0.0)	4 (29.9)	0 (0.0)	0 (0.0)	1 (11.2)	5 (40.3)	0 (0.0)	0 (0.0)	7 (57.5)	0 (0.0)	0 (0.0)	0 (0.0)	
*Personal care product manufacturing group *															
Herbal cosmetics manufacturers	65	13 (20.6)	24 (36.2)	6 (8.5)	6 (8.5)	7 (10.3)	10 (15.9)	19 (29.1)	0 (0.0)	6 (8.5)	5 (7.1)	0 (0.0)	6 (8.8)	7 (10.3)	**<0.0001**
Herbal sanitizer manufacturers	21	0 (0.0)	0 (0.0)	11 (50.0)	0 (0.0)	0 (0.0)	0 (0.0)	0 (0.0)	0 (0.0)	0 (0.0)	11 (50.0)	11 (50.0)	0 (0.0)	0 (0.0)	

*Note*. The questions were answered by companies that had experience with market globalization. The questions allowed for multiple answers. The values are the numbers (percentages) of participants.

^*∗*^ *P* value using the chi-squared test or Fisher's exact test for categorical variables; *P* values with statistical significance are presented in bold.

**Table 5:** Strategies cited by respondents for globalization of the Korean herbal market.

Manufacturers	Company no.	Establishment of overseas branches	Financial support	Improvement of intellectual property rights protection	Participation in international events	Assistance with foreign market surveys	Connections with overseas buyers	Seeking business partners	Acquiring foreign market information	Providing standard document templates	Assistance with document translation	Other
*Dietary supplement manufacturing group *												
Ginseng product manufacturers	186	31 (16.9)	57 (30.5)	3 (1.7)	16 (8.5)	66 (35.6)	13 (6.8)	110 (59.3)	38 (20.3)	6 (3.4)	3 (1.7)	6 (3.4)
Herbal drink manufacturers	108	29 (27.0)	38 (35.1)	3 (2.7)	17 (16.2)	6 (5.4)	20 (18.9)	44 (40.5)	12 (10.8)	6 (5.4)	6 (5.4)	6 (5.4)
Herb extract manufacturers	64	8 (12.5)	32 (50.0)	8 (12.5)	8 (12.5)	16 (25.0)	0 (0.0)	8 (12.5)	8 (12.5)	16 (25.0)	0 (0.0)	0 (0.0)
*Herbal medicine manufacturing group *												
Traditional herbal medicine manufacturers	111	12 (11.1)	37 (33.3)	12 (11.1)	12 (11.1)	0 (0.0)	37 (33.3)	49 (44.4)	37 (33.3)	0 (0.0)	12 (11.1)	0 (0.0)
Conventional herbal medicine manufacturers	12	0 (0.0)	5 (40.0)	2 (20.0)	0 (0.0)	7 (60.0)	0 (0.0)	5 (40.0)	0 (0.0)	0 (0.0)	0 (0.0)	0 (0.0)
*Personal care product manufacturing group *												
Herbal cosmetics manufacturers	65	6 (9.1)	12 (18.2)	6 (9.1)	18 (27.3)	0 (0.0)	18 (27.3)	18 (27.3)	19 (29.3)	6 (9.1)	0 (0.0)	0 (0.0)
Herbal sanitizer manufacturers	21	0 (0.0)	0 (0.0)	0 (0.0)	21 (100.0)	0 (0.0)	0 (0.0)	11 (50.0)	0 (0.0)	0 (0.0)	0 (0.0)	0 (0.0)

*Note*. The questions allowed for multiple answers. The values are the numbers (percentages) of participants.

## Abbreviations

R&D: Research and development
TKM: Traditional Korean medicine
KSIC-9: The ninth Korean Standard Industrial Classification
OTC: Over-the-counter
HDS: Herbal and dietary supplements
KIOM: Korea Institute of Oriental Medicine
WHO: World Health Organization
MFDS: Ministry of Food and Drug Safety
RDA: Rural Development Administration
KFS: Korea Forest Service
ICCMR: International Congress on Complementary Medicine Research
ODA: Official Development Assistance
SME: Small and medium-sized enterprise.
