# Supplementary material for: Perspectives on the Market Globalization of Korean Herbal Manufacturers: A Company-Based Survey
Source: Evid Based Complement Alternat Med. 2015 Jun 14;2015:515328. doi: 10.1155/2015/515328 (PMC4496648; doi:10.1155/2015/515328)
Supplement: Supplementary file 1 — Additional file 1: Questionnaire on the perceptions of market expansion and globalization. The survey instrument was designed to include two major sections. One section consisted of the questions regarding the characteristics of workforce. The other section consisted of the questions regarding the perception on the market expansion and strategies for market globalization. Additional file 2: Herbal items produced by respondent manufacturers. The respondents were asked what herbal species were commonly used as product sources. Red ginseng was predominant in ginseng product manufacturers, herbal drink manufacturers, and herb extract manufacturers. Additional file 3: Fifteen representative Korean herbal manufacturers' perceptions of market globalization. Forty percent of respondents in the large dietary supplementary manufacturer group cited “inexperience in administrative tasks” as the highest-rated limitation for market globalization. Meanwhile, the ratio for citing “Seeking business partners” as the challenging factor was either similar or a bit higher. [file 515328.f1.pdf]

Additional file 1. Market expiation questionnaire

한국한의학연구원
KOREA INSTITUTE OF ORIENTAL MEDICINE

- Please complete the following questions to reflect your opinions as accurately as possible.
- We thank you for taking the time to complete this questionnaire. Your answers and comments will be kept strictly confidential.

Market expansion questionnaire

- The purpose of this interview is to gather information on the general perception regarding market globalization among herbal manufacturing industry employees.
- This survey is only for scientific purposes.
- This questionnaire has been approved by the ethical review committee of the Korea Institute of Oriental Medicine (KIOM).

Serial number	Examiner name	Contact information
		( ) -

COMPANY INFORMATION	
(1) Type of legal organization	□ Individual proprietorship<br>□ Incorporated company<br>□ Non-business corporation<br>□ Government<br>□ Unincorporated association
(2) Type of establishment	□ Single-unit establishment (defined as a firm having no additional head office, agent office, business office, branch office, or branch shop)<br>□ Factory, agent office, business office, branch office and branch shop (defined as business units controlled by a single head office or by a central organization)<br>□ Head office or central organization (defined as business organizations that preside over more than one unit at different sites, such as factories, agent offices, business offices, branch offices, that solely perform management functions)

(3) Type of manufacturer	**Dietary supplement manufacturing group:**<br>□ **Ginseng product manufacturers** (defined as a manufacturer that produces ginseng goods using raw or processed ginseng materials)<br>□ **Herbal drink manufacturers** (defined as a manufacturer that produces tea or alcoholic or non-alcoholic beverages using herb materials)<br>□ **Herb extract manufacturers** (defined as a manufacturer that produces crude additives, liquefied additives, or health functional foods by processing natural herbs)<br><br>**Herbal medicine manufacturing group:**<br>□ **Traditional herbal medicine manufacturers** (defined as a manufacturer that produces standardized herbal medicines or herbal remedies on the basis of traditional Korean knowledge and literature)<br>□ **Conventional herbal medicine manufacturers** (defined as a manufacturer that produces labeled herbal medicines by processing a raw herb or its dried extracts into different forms, including tablets, capsules, syrups, essential oils, and ointments)<br><br>**Personal care product manufacturing group:**<br>□ **Herbal cosmetics manufacturers** (defined as a manufacturer that produces beauty goods, such as perfumes, beauty washes, cosmetics, or related products.<br>□ **Herbal sanitizer manufacturers** (defined as a manufacturer that produces a variety of sanitary items, including detergents, soaps, toothpastes, or cleansers, by adding herbal ingredients)<br><br>Other (please specify)<br>____

EMPLOYEE INFORMATION

(4) Occupational status	Number of workers			Annual wage (Unit: KW)					
	Male	Female	Total	100 billion	10 billion	1 billion	100 million	10 million	1 million
Regular permanent worker									
Temporary daily worker									
Self-employed worker									
Unpaid family employee									
Fixed-term employee									
Other									

(5) Occupational type	Researcher	Production worker	Office worker and others	Sum (A+B+C)
Male				
Female				

GENERAL INFORMATION

(6) Where does your company sell herbal products? Please rank the top two locations.

( ) Hospitals/Clinics

( ) Retailers/Wholesalers

( ) Chain stores/Direct outlets

( ) Internet/Online shopping

( ) Other (please specify)

(7) Which source materials do you typically use? Please mark all possible answers.

□ Extracts

□ Complete products

□ Semi-manufactured or intermediate products

□ Minimally processed products (washed, dried, or cut)

□ Raw materials (e.g., juice concentrate, powder, oils)

□ Other (please specify) ____________________

(8) Brand manufacturing		
What is your company's brand type? Please mark all possible answers and indicate each proportion.		
(1) Original brand manufacturer	(2) Original equipment manufacturer (OEM) or original design manufacturer (ODM)	(3) Joint brand or collective brand manufacturer
(%)	(%)	(%)

(9) Major export markets (country)		
Indicate the largest export markets for your products and the proportions.		
**First**	**Second**	**Third**
Country ( )<br>(%)	Country ( )<br>(%)	Country ( )<br>(%)

(10) Research environment		
Does your company have a research organization?		
□ Yes	□ No	□ I don't know
(11) If "yes", how much does your company annually invest in research?		
□ less than 100 million KW	□ 100 million KW ~ 1 billion KW	
□ 1 billion KW ~ 10 billion KW	□ more than 10 billion KW	

(12) Marketing environment					
Does your company have a marketing organization?					
□ Yes	□ No	□ I don't know			
(13) If "yes", does your original marketing strategy address international needs?					
□ Agree strongly	□ Agree slightly	□ Neutral	□ Disagree slightly	□ Disagree strongly	□ I don't know

EMPLOYEE'S PERCEPTION

(14) Which is the most important factor for the development of Korea's herbal industries? Please mark all possible answers.

□ Advertisement/public relations
□ Political support
□ Trust-building
□ Legislative support
□ R&D investment
□ Other (please specify)

(15) Does your company have global marketing experience?

□ Yes
□ No
□ I don't know

- (16) If "yes", what do you think is the primary limitation of global marketing? Please mark all possible answers.

□ Language problems
□ Difficulties of foreign trade processes
□ Business management
□ Political support
□ Low brand power
□ Lack of overseas market information
□ Lack of overseas branches
□ Low manpower
□ Insufficient funds
□ Inexperience in administrative tasks
□ Ignorance of overseas buyer demands
□ Lack of marketing strategies
□ Difficulties finding business partners
□ Other (please specify) ____________________

(17) What do you think is the most important factor for the globalization of Korea's herbal markets? Please mark all possible answers.

□ Acquiring foreign market information
□ Providing standard document templates
□ Assistance with document translation
□ Participation in international events
□ Establishment of overseas branches
□ Seeking business partners
□ Connection with overseas buyers
□ Improvement of intellectual property right protection
□ Assistance with foreign market surveys
□ Financial support
□ Other (please specify) ____________________

Additional file 2. Herbal items produced by respondent manufacturers

Manufacturers	Herbs or herbal ingredients primarily used by respondent manufacturers				
Ginseng product manufacturers	Red ginseng (*Hongsam*) 90.0%	Wild ginseng (*Sansam*) 1.8%	Fresh ginseng (*Susam*) 1.8%	Black ginseng (*Heuksam*) 1.4%	White ginseng (*Baeksam*) 0.9%
Herbal drink manufacturers	Red ginseng (*Hongsam*) 18.2%	*Rubus coreanus* (*bokbunja*) 16.9%	*Omija* 10.4%	Cornelian Cherry (*Sansuyu*) 7.8%	Acanthopanax (*Ogapi*) 6.5%
Herb extract manufacturers	Red ginseng (*Hongsam*) 20.0%	Aloe 8.0%	*Hovenia dulcis* 8.0%	Wild ginseng (*Sansam*) 8.0%	Gastrodia elata (*Chunma*) 8.0%
Traditional herbal medicine manufacturers	*Rehmanniae radix* (*Jiwhang*) 12.8%	Red ginseng *(Hongsam*) 10.6%	Glycyrrhiza (*Gamcho*) 10.6%	*Angelicae gigantis Radix* (*Danggwi*) 10.6%	*Puerariae radix* 8.5%

Additional file 3. Fifteen representative Korean herbal manufacturers' perceptions of market globalization

A. Respondents from large manufacturers cited difficulties experienced during preparations for global marketing

Large manufacturers*	Company No.	Inexperience in administrative tasks	Low manpower	Low brand power	Limitation of business management	Language problems	Lack of overseas market information	Lack of overseas branches	Difficulties with foreign trade processes	Insufficient funds	Lack of marketing strategies	Difficulties in finding business partners	Ignorance of overseas buyer demands	Other
**Dietary supplement manufacturing group**	10	4 (40.0)	3 (30.0)	1 (10.0)	-	-	1 (10.0)	2 (20.0)	-	1 (10.0)	2 (20.0)	3 (30.0)	-	-
**Herbal medicine manufacturing group**	5	-	-	2 (40.0)	-	-	-	2 (40.0)	-	-	3 (60.0)	-	1 (20.0)	-

*In this study, large manufacturers include manufacturers that employ more than 50 workers.

B. Respondents from large manufacturers cited strategies for globalizing the Korean herbal market

Large manufacturers*	Company No.	Establishment of overseas branches	Financial support	Improvement of intellectual property rights protection	Participation in international events	Assistance with foreign market surveys	Connections with overseas buyers	Seeking business partners	Acquiring foreign market information	Providing standard document templates	Assistance with document translation	Other
**Dietary supplement manufacturing group**	10	1 (10.0)	1 (10.0)	-	1 (10.0)	1 (10.0)	1 (10.0)	6 (60.0)	2 (20.0)	1 (10.0)	-	-
**Herbal medicine manufacturing group**	5	-	1 (20.0)	-	-	2 (40.0)	-	2 (40.0)	1 (20.0)	-	1 (20.0)	-

*In this study, large manufacturers include manufacturers that employ more than 50 workers.
